# Psychopharmacological treatment of anxiety and depression in patients with diabetes. Pharmacodynamics and pharmacokinetic considerations, drug interactions

**DOI:** 10.1192/j.eurpsy.2025.175

**Published:** 2025-08-26

**Authors:** F. Novais, D. Telles-Correia

**Affiliations:** Faculdade de Medicina, Lisboa, Portugal

## Abstract

**Abstract:**

Anxiety and depression are prevalent mental health conditions in patients with diabetes, significantly impacting their quality of life and complicating glycaemic control. The interplay between psychopharmacological agents and diabetes medications presents unique challenges in managing both mental health and metabolic conditions. This presentation reviews of psychotropic drugs commonly used to treat anxiety and depression in diabetic patients, with a focus on their efficacy, safety, and potential adverse effects. Special attention will be given to drug interactions between antidepressants, anxiolytics, and antidiabetic medications, which can influence treatment outcomes. Key considerations include the effects of psychotropic agents on insulin sensitivity, glucose metabolism, and the risk of hypoglycaemia. The presentation will also discuss personalized treatment strategies, adjusting for individual patient profiles and comorbidities. By integrating both psychiatric and endocrinological perspectives, we aim to improve clinical outcomes through optimized pharmacological management and minimize the risk of adverse drug interactions in this vulnerable patient population.

**Disclosure of Interest:**

None Declared

